# Improved electron collimation system design for Elekta linear accelerators

**DOI:** 10.1002/acm2.12155

**Published:** 2017-08-12

**Authors:** Garrett M. Pitcher, Kenneth R. Hogstrom, Robert L. Carver

**Affiliations:** ^1^ Department of Physics and Astronomy Louisiana State University Baton Rouge LA USA; ^2^ Mary Bird Perkins Cancer Center Baton Rouge LA USA

**Keywords:** electron collimation, electron Monte Carlo, leakage dose, therapeutic electron beams

## Abstract

Prototype 10 × 10 and 20 × 20‐cm^2^ electron collimators were designed for the Elekta Infinity accelerator (MLCi2 treatment head), with the goal of reducing the trimmer weight of excessively heavy current applicators while maintaining acceptable beam flatness (±3% major axes, ±4% diagonals) and IEC leakage dose. Prototype applicators were designed initially using tungsten trimmers of constant thickness (1% electron transmission) and cross‐sections with inner and outer edges positioned at 95% and 2% off‐axis ratios (OARs), respectively, cast by the upstream collimating component. Despite redefining applicator size at isocenter (not 5 cm upstream) and reducing the energy range from 4–22 to 6–20 MeV, the designed 10 × 10 and 20 × 20‐cm^2^ applicator trimmers weighed 6.87 and 10.49 kg, respectively, exceeding that of the current applicators (5.52 and 8.36 kg, respectively). Subsequently, five design modifications using analytical and/or Monte Carlo (MC) calculations were applied, reducing trimmer weight while maintaining acceptable in‐field flatness and mean leakage dose. Design Modification 1 beveled the outer trimmer edges, taking advantage of only low‐energy beams scattering primary electrons sufficiently to reach the outer trimmer edge. Design Modification 2 optimized the upper and middle trimmer distances from isocenter for minimal trimmer weights. Design Modification 3 moved inner trimmer edges inward, reducing trimmer weight. Design Modification 4 determined optimal X‐ray jaw positions for each energy. Design Modification 5 adjusted middle and lower trimmer shapes and reduced upper trimmer thickness by 50%. Design Modifications 1→5 reduced trimmer weights from 6.87→5.86→5.52→5.87→5.43→3.73 kg for the 10 × 10‐cm^2^ applicator and 10.49→9.04→8.62→7.73→7.35→5.09 kg for the 20 × 20‐cm^2^ applicator. MC simulations confirmed these final designs produced acceptable in‐field flatness and met IEC‐specified leakage dose at 7, 13, and 20 MeV. These results allowed collimation system design for 6 × 6–25 × 25‐cm^2^ applicators. Reducing trimmer weights by as much as 4 kg (25 × 25‐cm^2^ applicator) should result in easier applicator handling by the radiotherapy team.

## 1 INTRODUCTION

Our cancer center has seven Elekta Infinity radiotherapy accelerators (MLCi2 treatment head) with custom electron beams spanning 7–20 MeV (R_90_ values of 2.0, 2.5, 3.0, 3.5, 4.0, 5.0, and 6.0 ± 0.1 cm) and having slightly modified scattering foils.^1^, ^2^ Their in‐field (depth dose and beam flatness) and out‐of‐field (leakage) dose distributions are well suited for radiotherapy; however, our clinic feels there is opportunity for improved delivery technology by reducing electron applicator weights. Table 1 shows Elekta applicators are considerably heavier than comparable Varian applicators, primarily due to their trimmer weights.

**Table 1:** Applicator weights (kg) for Elekta Infinity and Varian 21EX

Applicator size (cm × cm)	Elekta weight (kg)		Varian weight (kg)	
	Total	Trimmers only	Total	Trimmers only
6 × 6	7.0	4.75	5.7	4.20^b^
10 × 10	7.7	5.52	6.5	5.00^b^
14 × 14	9.1	6.71	7.6^a^	6.10^a^^,^ b
20 × 20	10.9	8.36	8.6	7.10^b^
25 × 25	13.4	10.00	9.5	8.00^b^

a Weights are for 15 × 15‐cm^2^ applicator.

b Varian trimmer weights were estimated as the measured full applicator weight minus 1.5 kg.

Therefore, this study's purpose was to develop a methodology for designing electron collimation that produces Elekta applicators weighing no more than comparable Varian applicators. We have produced X‐ray jaw settings and trimmer designs for a set of five applicators (6 × 6, 10 × 10, 14 × 14, 20 × 20, and 25 × 25 cm^2^ at isocenter), based on the design process of 10 × 10 and 20 × 20‐cm^2^ applicators for 6–20 MeV beams. Our design for the Elekta MLCi2 treatment head should also apply to the newer Agility treatment head with only slight modifications, although that evaluation was not part of this study.

The criteria for suitability of collimator designs were that (a) applicator trimmer weights meet our design goals, (b) in‐field beam flatness criteria^3^ are met, and (c) out‐of‐field leakage dose meet IEC specifications.^4^ The criteria used to evaluate in‐field beam uniformity was that described by Hogstrom^1^, ^3^ for which off‐axis dose should not vary from central‐axis dose by more than ±3% along major axes (±4% along diagonals) in a region contained within 2 cm of the field edge (2√2 cm along diagonals). IEC states that mean dose in the leakage region, measured at 1‐cm depth along the major and diagonal axes from 4 cm outside field edge to M_10_ (geometric projection of primary collimator ∪ 10 cm outside field edge), should not exceed an energy‐dependent value (1.0%–1.8% of maximum central‐axis dose, D_max_). In addition, maximum dose in this region, measured along major and diagonal axes from 2 cm outside the field edge to the geometric projection of M_10_, should not exceed 10.0% of D_max_.^4^

The present work utilizes both in air, pencil‐beam dose calculations (Huizenga and Storchi^5^) and MC dose calculations^1^ for the Elekta Infinity. The in‐field pencil‐beam dose calculations have been validated by Pitcher,^2^ and the MC calculations for the Elekta beam model have been validated by Pitcher et al^1^ for beam energies of 7, 13, and 20 MeV. The MC dose calculations serve as both a design tool and one to confirm final collimation design.

## 2 METHODS

### 2.A Collimation system design specifications

This study designed a new Elekta electron collimation system, which like the current one, has five applicators (6 × 6, 10 × 10, 14 × 14, 20 × 20, and 25 × 25 cm^2^). The new collimation system specified field sizes at isocenter unlike the current system, which specifies them at the final trimmer position (5 cm above isocenter). This adjustment decreased trimmer weights by approximately 3%.

Also, the new collimation system was designed for 6–20 MeV electron beams, a reduction from 4 to 22 MeV currently allowed by Elekta. Any treatment requiring an energy less than 6 MeV can be treated with a 6‐MeV beam and bolus.^6^ Decreasing 22–20 MeV was justified by the increase in R_90_ with increasing E_p,0_ being small at energies greater than 20 MeV.^7^ This reduction allowed narrower and thinner trimmers, reducing applicator trimmer weights by approximately 30%.

Based on initial MC studies,^2^ three design parameters were established and maintained throughout the collimation system initial design process. First, tungsten was used for all applicator trimmers, based on their producing less leakage dose than copper or lead trimmers. Second, trimmers were designed 0.53‐cm (9.6‐g cm^−2^) thick, which reduced electron dose in water immediately distal to the trimmer to 1% of D_max_ with no shielding present for a 20‐MeV beam. Third, the upper and middle trimmer inner edges were aligned with beam divergence, which reduced leakage dose outside the field. Also, the lower trimmer inner edge divergence angle had little impact on leakage dose, allowing them to be parallel to central axis, consistent with inner edges typical of patient‐specific Cerrobend inserts.

### 2.B Initial design

The initial design used a method based on shielding primary electron dose,^8^, ^9^ for which inner and outer trimmer edges intercepted the penumbra from the upstream collimating component (95% and 2% of central‐axis dose, respectively) for the lowest beam energy (6 MeV). Thus, X‐ray and collimator‐scattered electron doses were ignored. The collimating components consisted of X‐ray jaws and upper, middle, and lower applicator trimmers, whose downstream trimmer surfaces were located at *z*‐positions of 70, 80, and 95 cm, respectively (73.3, 86.2, and 95 cm, respectively, for current Elekta 10 × 10 and 20 × 20‐cm^2^ applicators). The resulting applicator designs had trimmers weighing 6.87 and 10.49 kg, respectively, exceeding those of current Elekta applicators (5.52 and 8.36 kg, respectively) and our objective (5.00 and 7.10 kg, respectively).

Hence, five modifications were made to the initial designs to reduce trimmer weights to acceptable levels, while maintaining acceptable in‐field flatness and out‐of‐field leakage dose. These modifications, which focused on 10 × 10 and 20 × 20‐cm^2^ applicators, included (a) beveling outer trimmer edges, (b) optimizing upper and middle trimmer *z*‐positions, (c) determining inner trimmer edge positions, (d) determining X‐ray jaw positions for each energy, and (e) determining thicknesses and bevel shapes of each trimmer.

### 2.C Design modification 1—Beveling outer trimmer edges

The trimmer's outer cross‐section, referred to as the “outer trimmer edge”, was beveled, matching the thickness required to stop electrons (1% transmission) to off‐axis position of the 2% fluence off‐axis ratio (OAR) of each beam energy (E). These off‐axis positions, located 2.05*σ*_*x*_ outside the projection of the upstream collimator's inner edge, were determined for energies 6–20 MeV (1‐MeV spacings). The OAR is given by

(1) $$OAR\left(E,x\right)=0.5\left[1-erf\left(\frac{x}{\sqrt{2}\sigma_{x}\left(E\right)}\right)\right],$$

where x is the distance from the projection of the inner edge of the upstream collimating component at the position of the downstream collimating component. Sigma of the Gaussian in the *x*‐*z* plane, *σ*_*x*_ *(E)*, for electron pencil beams originating at the *x‐y* plane of the upstream collimating component (*z’*) and arriving at the *x‐y* plane of the downstream collimating component (*z*) is given by^8^, ^9^

(2) $$\sigma_{x}^{2}\left(E\right)=T_{air}\left(E\right)\cdot \left(z-z^{\prime }\right)\cdot \left(\frac{z}{6}\right)+\sigma_{SW}^{2}\left(E\right){\left(\frac{z-z^{\prime }}{z^{\prime }}\right)}^{2},$$

where *z’* and *z* represent central‐axis distances from the virtual source position to the position of the upstream and downstream collimating components, respectively, and *T*_*air*_ *(E)* is the electron scattering power in air.

A virtual source position 94 cm from isocenter, used for all beam energies, was determined at 7 MeV using Schröder‐Babo methodology.^10^ *σ*_*SW*_ *(E)*, sigma of the Gaussian virtual source width, was determined from eq. (2), using *σ*_*x*_ *(E)* of measured profiles and calculation of the air‐scatter term using, *T*_*air*_ *(E) = 0.00554·E* ^*−1.78*^, based on the 10‐MeV ICRU 35^11^ value and Werner's approximation.^12^ The resulting value of 2.0 cm for *σ*_*SW*_ was used for all energies.

**Figure 1:**
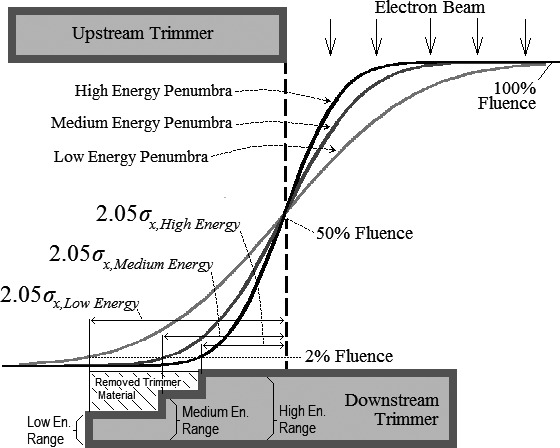
Illustration of design method for beveled outer trimmer edge. Calculated penumbras cast by upstream onto downstream trimmer are plotted for three energies. The low energy 95% OAR falls at the downstream trimmer inner edge; the 50% OAR falls at the geometric projection of the upstream trimmer; and 2% OAR is shown for each of the three energies. The material thickness (range) required to stop electrons at each energy is indicated, extending to the off‐axis position of the 2% OAR. This reduces trimmer thickness towards the outer edge (cross‐hatched area), creating a beveled shape and lighter trimmer.

Figure 1 illustrates the effect of this modification on trimmer shape, showing narrow, medium, and broad penumbras for high, medium, and low‐energy electron beams, respectively. The lateral trimmer width decreases with increasing energy, reducing trimmer cross‐sectional area, and hence weight.

### 2.D Design modification 2—Optimizing upper and middle trimmer *z*‐positions

Design Modification 2 optimized *z*‐positions of the upper and middle trimmers to minimize 10 × 10 and 20 × 20‐cm^2^ applicator weights. Combinations of the upper and middle trimmer *z*‐positions were varied (0.5‐cm step size) from 55 to 93 cm with at least 2 cm separation. For each combination, trimmer shapes (inner edge positions and outer bevels) were determined according to Design Modification 1.

### 2.E Design modification 3—Determining inner trimmer edge positions

In Design Modification 3, the OAR at which the inner edge is positioned (inner edge OAR) was reduced from 95% (value in Design Modification 2) for each trimmer. This moved each upstream collimator's inner edge toward central axis, which reduced each upstream trimmer's length and each downstream trimmer's width, reducing trimmer weights. OARs were reduced as much as possible, while maintaining acceptable in‐field flatness. Determining optimal inner edge fluence matching OARs was performed in two steps, first using analytical pencil‐beam calculations and second using MC calculations.

#### 2.E.1 Step 1—Optimizing using analytical electron beam model

A five‐dimensional search grid optimization, which minimized applicator trimmer weights while maintaining acceptable in‐field beam flatness, was performed for the 10 × 10 and 20 × 20‐cm^2^ applicator designs. Inner edge OARs for each of the three trimmers were reduced from previously used 95%, and upper and middle trimmer *z*‐positions were varied.

Optimization was performed in three iterations. Each iteration's optimal design started the next iteration, and parameter step sizes decreased with each iteration. Table 2 shows approximate upper and lower boundaries and step sizes for each of the five parameters. All other design parameters, including bevel shape calculation parameters, were maintained.

**Table 2:** Example upper and lower boundaries and grid step sizes of the five parameters for the three iterations of the search grid optimization are written as “lower boundary‐upper boundary (step size)”. Seach grid values differed slightly for 10 × 10 and 20 × 20‐cm^2^ applicators

Design parameters	Trimmer	First iteration	Second iteration	Third iteration
Inner edge matching OAR (%)	Upper	87.5–95.0% (1.5%)	84.0–89.0% (1.0%)	83.5–86.0% (0.5%)
	Middle	86.0–95.0% (1.5%)	87.0–92.0% (1.0%)	88.5–91.0% (0.5%)
	Lower	86.0–95.0% (1.5%)	88.5–93.5% (1.0%)	90.0–92.5% (0.5%)
Trimmer *z*‐position (cm)	Upper	58.5–64.5 (1.5)	62.5–66.5 (1.0)	64.5–66.5 (0.5)
	Middle	74.5–80.5 (1.5)	77.0–81.0 (1.0)	78.0–80.0 (0.5)

In‐field flatness was evaluated for each applicator design from OARs calculated using Huizenga and Storchi's^5^ analytical model of the primary electron beam, which uses scattering moment profiles to transport primary electron fluence profiles in air through multiple collimation levels (X‐ray jaws and three trimmers) to isocenter.^2^ Calculations were performed at 6 MeV, the lowest energy and that most likely to fail flatness due to having the greatest scattering power. Major axes OARs (in‐plane and cross‐plane) were considered acceptably flat if the OAR varied (decreased) from central‐axis dose by ≤2% at the edge of the uniformity region (2 cm inside field edge). The 2% threshold should ensure that the OAR did not vary from central‐axis value by >4%, the maximum variation allowed for acceptable diagonal profiles.^3^

#### 2.E.2 Step 2—Adjustments using MC calculations

When MC calculations revealed that the collimation system designed in Step 1 narrowly failed our flatness criteria,^3^ four modified designs for the 10 × 10 and 20 × 20‐cm^2^ applicators were created by incrementally increasing inner edge OARs produced from Step 1 optimizations, while maintaining trimmer *z*‐positions. This stepped trimmers and jaws outward from central axis, improving in‐field flatness while slightly increasing applicator weights. Table 3 lists increased OARs selected for the modified applicators, increasing weight fairly uniformly with each increment (approximately 0.4 and 0.15 kg for 10 × 10 and 20 × 20‐cm^2^ applicators, respectively).

**Table 3:** Inner edge OARs for each trimmer of 10 × 10 and 20 × 20‐cm^2^ applicators modelled for MC inner edge adjustment analysis. Design A represents result produced from search grid optimization using analytical model; Designs B‐E represent modified designs created by incrementally increasing inner edge OARs. Incremental increases in mass (Δm) are listed

Applicator designs	10 × 10‐cm^2^ Applicator				20 × 20‐cm^2^ Applicator			
	Upper trim.	Middle trim.	Lower trim.	Δm (kg)	Upper trim.	Middle trim.	Lower trim.	Δm (kg)
Design A	85.0%	90.0%	92.0%	–	85.0%	89.0%	92.0%	–
Design B	90.0%	92.0%	94.0%	0.43	86.0%	89.5%	92.5%	0.12
Design C	93.0%	94.0%	95.0%	0.36	87.0%	90.0%	93.0%	0.13
Design D	95.0%	96.0%	96.5%	0.47	89.0%	91.0%	93.0%	0.16
Design E	96.5%	97.5%	98.0%	0.58	90.0%	92.0%	93.5%	0.18

Each of these five applicator designs was modelled in BEAMnrc by inserting them into a model of our Elekta Infinity accelerator (MLCi2 treatment head).^1^, ^2^, ^13^ MC simulations with our lowest energy, 7‐MeV beam (E_p,0_ = 7.14 MeV, R_90_ = 2.0 cm) were performed to evaluate in‐field flatness. Resulting phase‐space files 1 cm upstream of isocenter were input into DOSXYZnrc, which calculated dose distributions in a water phantom at 100‐cm SSD. Dose was calculated in a matrix of 0.5‐cm, cubed voxels centered at 1‐cm depth (0.75–1.25 cm), from which major‐axis profiles in both the in‐plane and cross‐plane dimensions were symmetrized about central axis to improve statistical uncertainty and normalized to central‐axis dose (averaged over central 3 × 3 voxels). For each model of the 10 × 10 and 20 × 20‐cm^2^ applicators, those producing the least applicator trimmer weight, while maintaining acceptable in‐field flatness, were selected for continuing the collimation system design process.

### 2.F Design modification 4—Determining X**‐**ray jaw positions for each energy

**Figure 2:**
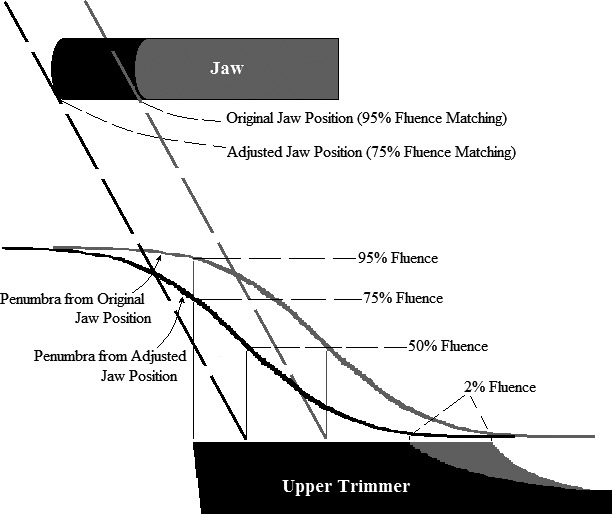
Illustration of effect of X‐ray jaw position adjustment on upper trimmer beveled outer edge. Shown are 20‐MeV penumbras cast by jaws, positioned such that the upper trimmer inner edge falls at 95% (original, shaded gray) and 75% (adjusted, shaded black) OARs. Geometric projections of diverging beam edges (50% OAR) are shown as gray (black) dashed lines for the original (adjusted) jaw position. Upper trimmer cross‐section is designed with 95% OAR (shaded gray) and 75% OAR (shaded black). For both designs, the off‐axis extension of full trimmer thickness matches the 2% OAR position at 20 MeV. The gray region demarcates material removed from trimmer by adjustment.

Design Modification 4 had two benefits, reducing both upper trimmer weight and out‐of‐field leakage dose. Upper trimmer weight was reduced by moving the X‐ray jaws toward central axis, which decreased the OAR value at the upper trimmer inner edges; this moved the outer edge bevel shape toward central axis, reducing its cross‐sectional area. This is illustrated in Fig. 2 for a 20 MeV beam, which shows cross‐sectional views of the upper trimmer and X‐ray jaw positioned for two different OAR values at the upper trimmer inner edge, as well as corresponding dose profiles in the penumbra cast by the X‐ray jaw.

At 20 MeV, the optimum design for each applicator (least trimmer weights and acceptable flatness and leakage) was selected from three designs, i.e., X‐ray jaw settings with OAR values at the upper trimmer inner edges of 95%, 75%, and 55% for the 10 × 10‐cm^2^ applicator and 89%, 72%, and 55% for the 20 × 20‐cm^2^ applicators. For intermediate energies (6–20 MeV), X‐ray jaw positions for 10 × 10 and 20 × 20‐cm^2^ applicators were determined by linearly interpolating between OAR values at the upper trimmer inner edges for 6 MeV (Design Modification 3) and those optimized for 20‐MeV.

To evaluate effects of adjusted X‐ray jaw positions and upper trimmer designs on in‐field flatness and out‐of‐field leakage dose, each of the three new 10 × 10 and 20 × 20‐cm^2^ collimation system designs were modelled and inserted into our Elekta Infinity BEAMnrc model, and MC calculations were performed at energies of 7, 13 and 20 MeV. Table 4 shows the resulting X‐ray jaw settings for each applicator. All source and transport parameters used in Modification 3 were maintained, and dose was calculated at depths of 1 and 2 cm in water. Within the field, dose profiles at 1‐cm depth at 7 MeV and 2‐cm depth at 13 and 20 MeV were used to assess in‐field flatness; also, dose profiles at 1‐cm depth were used to evaluate IEC mean percent leakage dose.

**Table 4:** Off‐axis X‐ray jaw positions (cm), expressed as “In‐Plane x Cross‐Plane” position projected to isocenter, for the three collimation system designs analyzed in Design Modification 4 for 7, 13, and 20 MeV beams. The three designs, “Unadjusted Jaw Positions” (results of Design Modification 3), “Jaw Adjustment A”, and “Jaw Adjustment B”, correspond to 95%, 75%, and 55% OAR values at the upper trimmer inner edge, respectively for the 10 × 10‐cm^2^ applicator, and to 89%, 72%, and 55%, respectively, for the 20 × 20‐cm^2^ applicator

Collimation design	7 MeV	13 MeV	20 MeV
10 × 10‐cm^2^ Collimator			
Unadjusted jaw positions	12.7 × 9.8	12.3 × 9.4	12.1 × 9.3
Jaw adjustment A	12.5 × 9.6	11.4 × 8.4	10.9 × 7.7
Jaw adjustment B	12.4 × 9.4	10.9 × 7.8	10.2 × 6.8
20 × 20‐cm^2^ collimator			
Unadjusted jaw positions	16.8 × 12.2	16.5 × 11.9	16.4 × 11.8
Jaw adjustment A	16.7 × 12.1	16.0 × 11.3	15.6 × 10.8
Jaw adjustment B	16.6 × 12.0	15.6 × 10.8	15.0 × 10.0

### 2.G Design modification 5—Determining trimmer thickness and bevel shape

Design Modification 5 reduced trimmer thickness (previously 1% primary electron transmission) and adjusted outer trimmer edge bevel shape. Their impact on reducing trimmer weight, while maintaining leakage specifications, was investigated using MC calculations for the 20 × 20‐cm^2^ applicator at 20 MeV. Resulting mean percent leakage doses were compared with trimmer weight to determine the best trimmer modifications for reducing applicator weight, while maintaining acceptable 20‐MeV leakage dose.

#### 2.G.1 Trimmer thickness reduction analysis

**Figure 3:**
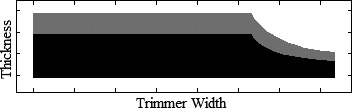
Design Modification 5: Trimmer Thickness Reduction. Trimmer designed by scaling full thickness (1% electron transmission) by 65% (black) reduces weight of trimmer designed with full thickness (black and gray) by 35% (gray).

The 20 × 20‐cm^2^ applicator from Design Modification 4 was modified one trimmer at a time (the other two maintaining full thickness); thickness was reduced by scaling the variable trimmer thicknesses by various percentages (c.f. Fig. 3). For the lower and middle trimmers, trimmer thicknesses were reduced by 7%, 14%, 21%, and 35%; for the upper trimmer, thicknesses were reduced by 7%, 14%, 21%, 35%, and 49%, producing 13 new applicator designs. BEAMnrc MC simulations were performed for each of the 13 20 × 20‐cm^2^ applicator designs at 20 MeV. Dose at 1‐cm depth in water was calculated using methodology detailed in Design Modification 3.

#### 2.G.2 Bevel shape adjustment

Modified 20 × 20‐cm^2^ applicator designs were created by adjusting trimmer bevel shapes from Design Modification 4, which were generated using a 2% OAR at the trimmer outer edges for 6–20 MeV beams. Modified trimmers were designed with OARs increased from 2% to 25% and 48% at 20 MeV; intermediate energy OARs at the trimmer outer edges were linearly interpolated between 20‐MeV values and the 2% OAR at 6 MeV. Again, the 20 × 20‐cm^2^ applicator from Design Modification 4 was modified one trimmer at a time (other two maintaining their original bevel shape), producing six modified applicator models.

**Figure 4:**
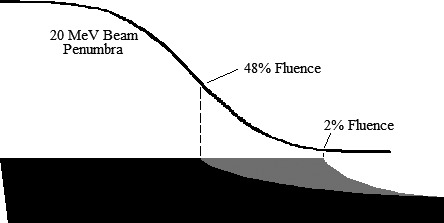
Design Modification 5: Bevel Shape Adjustment: 20‐MeV penumbra cast on a trimmer designed with two different outer edge bevel shapes. Constant inner thickness (1% electron transmission) is for 20‐MeV electrons; minimal outer thickness is for 6‐MeV electrons. Gray and black regions demarcate trimmer cross‐section when designed with tapered bevel with 2% OARs at outer edge for all energies. Black region demarcates the trimmer shape designed with a 48% OAR at outer edge for 20 MeV. Gray region demarcates trimmer reduction.

Increasing the 20‐MeV outer edge OARs pulled the outer edge bevel toward central axis while maintaining the full width of the trimmer (c.f. Fig. 4). Trimmer weight for each of the six modified applicators was calculated to evaluate the effect of the bevel shape adjustment. To determine the effect of these modifications on leakage dose, BEAMnrc simulations were performed for the six adjusted 20 × 20‐cm^2^ applicator designs at 20 MeV using our Elekta Infinity model. Dose at 1‐cm depth in water was calculated using the methodology detailed in Design Modification 3.

### 2.H Evaluating final collimation system designs

Following completion of Design Modification 5, 10 × 10 and 20 × 20‐cm^2^ applicator collimating system designs were inserted into our BEAMnrc Elekta Infinity model of the MLCi2 treatment head. MC calculations at 7, 13, and 20 MeV produced dose profiles at 1‐ and 2‐cm depths in water using methodology from Design Modification 3. These results were used to assess in‐field flatness and out‐of‐field, patient‐plane leakage dose of the final collimation system designs.

## 3 RESULTS

### 3.A Design modification 1—Beveling outer trimmer edges

**Figure 5:**
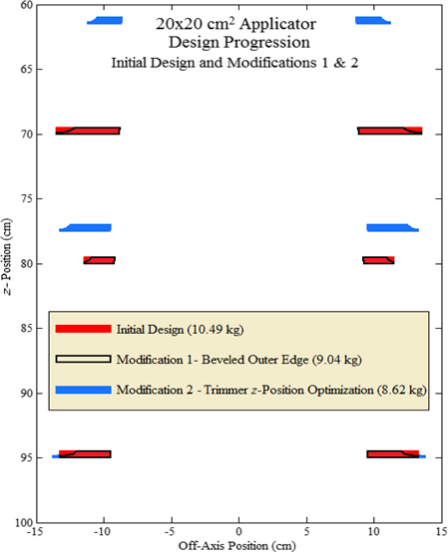
Results of Design Modifications 1 and 2. In‐plane cross‐sectional views of 20 × 20‐cm^2^ applicator trimmers for initial design (trimmers shaded red) with those from Design Modifications 1 and 2. Trimmers following Design Modification 1 (beveling outer trimmer edges) are outlined in black, and trimmers following Design Modification 2 (trimmer *z*‐position optimization) are shaded blue.

Figure 5, an in‐plane cross‐sectional scaled drawing of 20 × 20‐cm^2^ applicator trimmers, compares the initial design with the modified design whose beveled outer edges remove material, reducing trimmer weights. Table 5 lists trimmer weights and percent weight reductions following each design modification, showing 14.7% and 13.8% weight reductions for the 10 × 10 and 20 × 20‐cm^2^ applicators, respectively.

**Table 5:** Summary of weight reduction results for each step in collimation design process. For 10 × 10 and 20 × 20‐cm^2^ applicators, trimmer weights and corresponding percent weight reductions, both step‐to‐step (Mod.) and cumulative (Cum.), following each design modification are shown

	10 × 10‐cm^2^ applicator			20 × 20‐cm^2^ applicator		
	Trimmer weights (kg)	% Reduction		Trimmer weights (kg)	% Reduction	
		Mod.	Cum.		Mod.	Cum.
Initial design	6.87	–	–	10.49	–	–
Design modification 1—Bevel outer edges	5.86	14.7%	14.7%	9.04	13.8%	13.8%
Design modification 2—Trimmer height optimization	5.52	5.8%	19.7%	8.62	4.7%	17.8%
Design modification 3—Inner trimmer edge position Analysis	5.87	−6.3%	14.6%	7.73	10.3%	26.3%
Design modification 4—Beam energy dependence of jaw position analysis	5.43	7.5%	21.0%	7.35	4.9%	29.9%
Design modification 5—Trimmer thickness & outer bevel shape adjustment	3.73	31.3%	45.7%	5.09	30.8%	51.5%
Design goal	5.00	–	–	7.10	–	–

### 3.B Design modification 2—Optimizing upper and middle trimmer *z*‐positions

**Figure 6:**
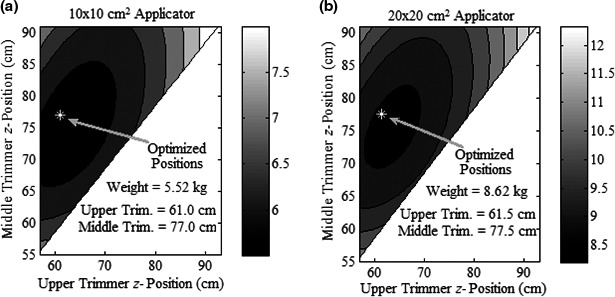
Results of Design Modification 2. Isomass (kg) contour plots of trimmers for (a) 10 × 10 and (b) 20 × 20‐cm^2^ applicators, plotted versus *z*‐positions of downstream surface of middle and upper trimmers. White stars demarcate trimmer *z*‐positions producing minimal mass of trimmers.

Results in Fig. 6 show isomass plots versus upper and middle trimmer *z*‐positions for the 10 × 10 and 20 × 20‐cm^2^ applicators. As the upper and middle trimmers become approximately equally spaced between the jaws and lower trimmer, total trimmer weight is minimized. Optimal *z*‐positions of the upper and middle trimmer downstream surfaces are 61.0 and 77.0 cm, respectively, for the 10 × 10‐cm^2^ applicator, and 61.5 and 77.5 cm, respectively, for the 20 × 20‐cm^2^ applicator. Figure 5 illustrates how the upper and middle trimmers moving upstream increases middle and lower trimmer widths slightly from the previous design, but decreases upper trimmer width, causing net trimmer weight reductions of 5.8% and 4.7% for 10 × 10 and 20 × 20‐cm^2^ applicators, respectively (c.f. Table 5).

### 3.C Design modification 3—Determining trimmer inner edge positions

**Figure 7:**
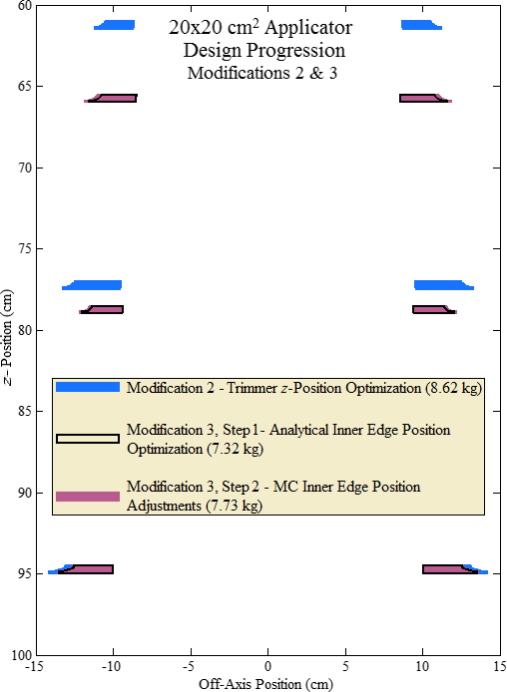
Results of Design Modifications 2 and 3. In‐plane cross‐sectional view of 20 × 20‐cm^2^ applicator trimmers showing progression from Design Modification 2 to 3. Trimmers resulting from Design Modification 2 shaded blue; trimmers from Design Modification 3, Step 1 (analytical‐calculated inner edge OAR reduction and *z*‐position optimization) outlined in black; and trimmers from Design Modification 3, Step 2, (MC‐calculated inner edge OAR adjustments) shaded violet.

Optimization of collimation inner edge positions using our analytical model (Design Modification 3, Step 1) reduced upper, middle, and lower trimmers inner edge OARs from 95% to 85%, 90%, and 92%, respectively, for the 10 × 10‐cm^2^ applicator and to 85%, 89%, and 92%, respectively, for the 20 × 20‐cm^2^ applicator. In addition, *z*‐positions of upper and middle trimmers (downstream surfaces) were optimized to 66 and 79 cm, respectively. Figure 7 compares the resulting in‐plane cross‐sectional view of the 20 × 20‐cm^2^ applicator trimmers with that from Design Modification 2, illustrating how the optimization moved upper and middle trimmers toward central axis. This reduced the 10 × 10 and 20 × 20‐cm^2^ applicator trimmer weights to 4.61 and 7.32 kg, respectively.

**Figure 8:**
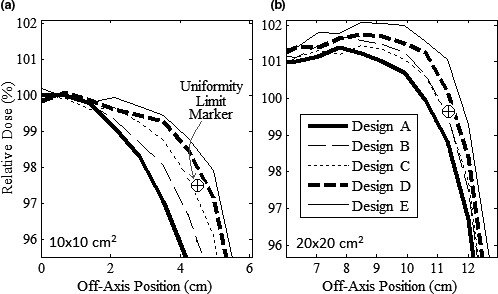
Results of Design Modification 3, Step 2 (inner edge adjustment). In‐field diagonal profiles of MC‐calculated relative dose versus off‐axis position (1‐cm depth in water) at 7 MeV for (a) 10 × 10 and (b) 20 × 20‐cm^2^ applicators. Designs A→E increased inner edge OARs according to Table 3. The Uniformity Limit Marker “⊕” indicates minimum dose at edge of uniformity region (2√2 cm inside field corner) required for in‐field flatness.

However, MC calculations showed designs using the analytical optimization marginally failed in‐field flatness for 10 × 10 and 20 × 20‐cm^2^ applicators (Design A, Fig. 8). Therefore, Design Modification 3, Step 2 made fine adjustments to trimmer positions using Table 3 settings, incrementally increasing fluence matching OARs. Figure 8 compares diagonal profiles of MC‐calculated relative dose versus off‐axis position for 10 × 10 and 20 × 20‐cm^2^ applicators at 7 MeV for these adjustments. The uniformity limit markers in Fig. 8, which demarcate the minimum OAR (96%) for acceptable in‐field flatness at the edge of the uniformity region, have relative doses of 97.77% and 99.59% for the 10 × 10 and 20 × 20‐cm^2^ applicators, respectively. Differences from 96% arise from including a 1% cushion and having been corrected for differences between MC‐calculated and measured doses with the current clinical collimation system.^2^ In Fig. 8, Designs B and C, like Design A, produced unacceptably flat profiles for both applicators. Design D, having the smallest edge position adjustments (and weight increase) while producing an acceptably flat profile, was selected for proceeding with the design process.

Shown in Table 5 the resulting 20 × 20‐cm^2^ trimmer weights decreased 10.3% from results of Design Modification 2, whereas the 10 × 10‐cm^2^ trimmer weights increased 6.3%. The 20 × 20‐cm^2^ applicator design resulting from these slight trimmer inner edge position adjustments (Step 2 of Design Modification 3) is demarcated in Fig. 7.

### 3.D Design modification 4—Determining X**‐**ray jaw positions for each energy

**Figure 9:**
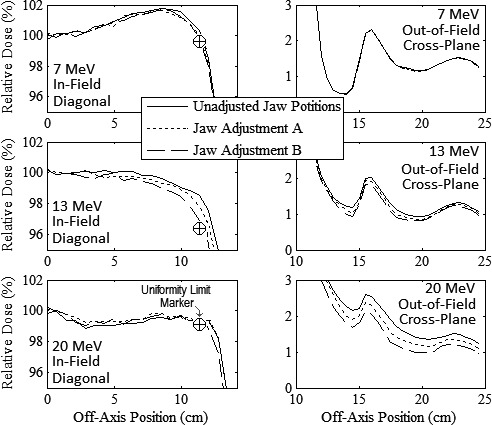
Impact of X‐ray Jaw Position, Design Modification 4. In‐field diagonal (left) and out‐of‐field cross‐plane (right) MC‐calculated relative dose versus off‐axis position profiles for 20 × 20‐cm^2^ collimation system designs at 7 (upper), 13 (middle), and 20 MeV (lower). Plots for three collimation system designs, “Unadjusted Jaw Positions”, “Jaw Position A”, “Jaw Position B” (c.f. Table 4), are shown. Profiles are normalized to central‐axis dose at calculation depths of 1 cm at 7 MeV and 2 cm at 13 and 20 MeV. The Unifomrity Limit Marker “⊕” indicates minimum dose required to meet in‐field flatness at edge of uniformity region.

MC‐calculated diagonal in‐field relative dose versus off‐axis position profiles for the three collimation system designs evaluated in Design Modification 4 are plotted in Fig. 9 for the 20 × 20‐cm^2^ applicator at 7, 13, and 20 MeV. The three designs, labelled “Unadjusted Jaw Positions”, “Jaw Adjustment A”, and “Jaw Adjustment B”, correspond to 20‐MeV OAR values at the upper trimmer inner edge dose profile of 89% (from Design Modification 3), 72%, and 55%, respectively. Results showed all three designs produced acceptably flat beams within the field at 7, 13, and 20 MeV. Not shown, MC calculations showed similar results for the 10 × 10‐cm^2^ applicator. Hence, a 55% OAR at the upper trimmer inner edge at 20 MeV was selected for proceeding with the collimation system design process. This modification adjusted the off‐axis position of the 20‐MeV in‐plane and cross‐plane X‐ray jaws from 5.1 to 4.8 cm (12.1 and 9.3 cm projected to isocenter) to 4.3 and 3.5 cm (10.2 and 6.8 cm projected to isocenter), respectively, for the 10 × 10‐cm^2^ applicator, and from 6.9 to 6.0 cm (16.4 and 11.8 cm projected to isocenter) to 6.3 and 5.1 cm (15.0 and 10.0 cm projected to isocenter), respectively, for the 20 × 20‐cm^2^ applicator. Table 5 shows these designs reduced trimmer weights by 7.5% and 4.9% for the 10 × 10 and 20 × 20‐cm^2^ applicators, respectively.

In addition, Fig. 9 results show that adjusting the jaws inward decreased out‐of‐field leakage dose. This effect increased with energy due to greater jaw adjustment, having negligible effect at 7 MeV. Compared to the “Unadjusted Jaw Positions” design, the “Jaw Adjustment B” design decreased the 20‐MeV beam mean percent leakage dose from 1.26% to 0.82% for the 10 × 10‐cm^2^ applicator and from 1.21% to 0.89% for the 20 × 20‐cm^2^ applicator.

### 3.E Design modification 5—Determining trimmer thickness and bevel shape

**Figure 10:**
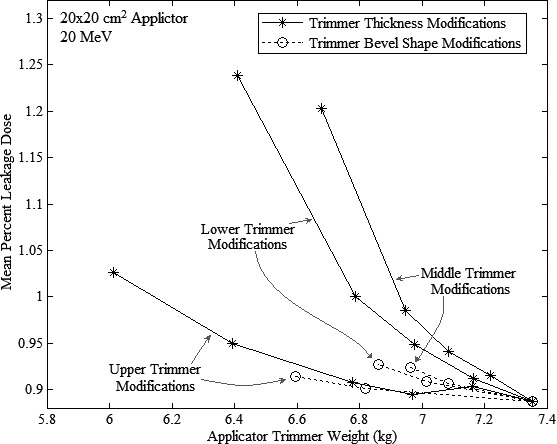
Results of Design Modification 5, MC‐calculated mean percent leakage dose (% of D_max_) versus applicator trimmer weight at 20 MeV for 20 × 20‐cm^2^ applicator. Designs for modified trimmer thickness reductions and bevel shape adjustments are dashed and solid curves, respectively. Curves representing modifications to upper, middle, and lower trimmers are labelled. Common point in lower right represents unmodified 20 × 20‐cm^2^ design (result of Design Modification 4).

Figure 10 shows results of trimmer thickness reductions and bevel shape adjustments, plotting MC‐calculated mean percent leakage dose versus applicator trimmer weight for the 20 × 20‐cm^2^ applicator at 20 MeV. Each of the six curves represents a unique trimmer‐modification combination, and the common, unmodified point representing the result of Design Modification 4. All six curves show increased mean leakage dose with decreased applicator trimmer weight with the more gradually sloped curves being the more beneficial modifications. The lower and middle trimmers show the bevel shape adjustments to be more beneficial than trimmer thickness reductions; hence, a 20‐MeV OAR for the outer bevel of 50% (48% rounded) was selected for design of the lower two trimmers. The upper trimmer showed both modifications similarly beneficial; however, applicator trimmer weight was decreased further by reducing trimmer thickness than bevel shape adjustment. Therefore, a 50% (49% rounded) thickness reduction was selected for the upper trimmer. Although only investigated for the 20 × 20‐cm^2^ applicator, these same modifications were implemented for the 10 × 10‐cm^2^ applicator, both resulting in applicator trimmer weight reductions of approximately 31% (c.f. Table 5).

### 3.F Evaluating final collimating system designs

**Figure 11:**
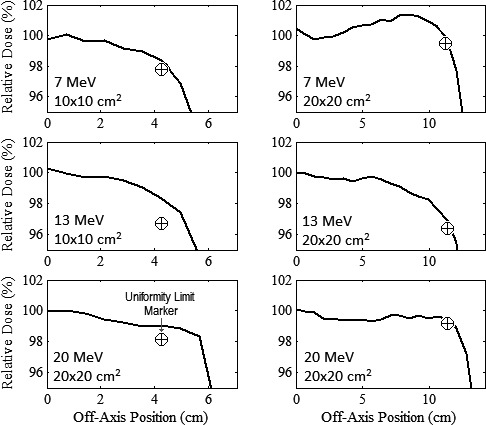
In‐field flatness of final collimation system design. Diagonal profiles of MC‐calculated relative dose versus off‐axis position are plotted for 10 × 10 (left) and 20 × 20‐cm^2^ (right) applicators with 7‐MeV profiles (upper row) at 1‐cm depth, and 13‐MeV (middle row) and 20‐MeV (lower row) profiles at 2‐cm depth in water. The Uniformity limit marker “⊕” demarcates minimum dose at edge of uniformity region for a profile to be acceptably flat.

Figure 11 plots profiles of in‐field MC‐calculated relative dose versus off‐axis position along the diagonal axis for the 10 × 10 and 20 × 20‐cm^2^ applicators at 7, 13, and 20 MeV. Because all profiles pass above the uniformity limit markers and do not exceed 103%, both collimation systems produced acceptable in‐field flatness along the diagonals. Similarly, in‐field dose profiles were acceptably flat along the major axes.^2^

**Figure 12:**
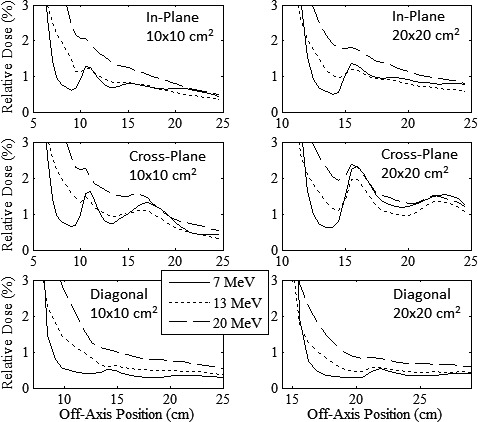
Out‐of‐field leakage dose for final collimation system designs. In‐plane (upper row), cross‐plane (middle row), and diagonal (lower row) plots of MC‐calculated relative dose versus off‐axis position for 10 × 10‐cm^2^ (left) and 20 × 20‐cm^2^ (right) applicators at 7 (solid curve), 13 (short‐dashed curve), and 20 MeV (long‐dashed curve). All profiles are normalized to central‐axis dose at 1‐cm water depth.

Figure 12 plots out‐of‐field profiles (in‐plane, cross‐plane, and diagonal) of MC‐calculated relative dose versus off‐axis position at 1‐cm depth for the new 10 × 10 and 20 × 20‐cm^2^ applicators at 7, 13, and 20 MeV. These results show for both applicators that the 20‐MeV beam has the greatest leakage dose, quantified by the IEC‐calculated mean percent leakage doses in Table 6. In addition, Table 6 data shows that mean percent leakage doses for 10 × 10 and 20 ×20‐cm^2^ applicators were less than IEC limits at 7, 13, and 20 MeV.

**Table 6:** MC‐calculated mean percent leakage doses (% of D_max_) per IEC specifications for final 10 × 10 and 20 × 20‐cm^2^ collimation system designs at 7, 13, and 20 MeV, compared with IEC‐specified maximum.^4^

Beam energy (MeV)	10 × 10‐cm^2^ applicator	20 × 20‐cm^2^ applicator	IEC‐specified maximum
7	0.58%	0.79%	1.00%
13	0.63%	0.75%	1.10%
20	1.03%	1.11%	1.34%

For the 10 × 10‐cm^2^ applicator, mean percent leakage doses at 7, 13, and 20 MeV were 0.58%, 0.63%, and 1.03% of D_max_, respectively, below IEC limits of 1.00%, 1.10%, and 1.34%, respectively. For the 20 × 20‐cm^2^ applicator mean percent leakage doses were 0.79%, 0.75%, and 1.11% of D_max_, respectively, also below IEC limits.

## 4 SUMMARY AND DISCUSSION OF RESULTS

### 4.A Summary of weight reduction in design process

10 × 10 and 20 × 20‐cm^2^ collimation systems for the Elekta Infinity (MLCi2 treatment head) were initially designed with (a) inner and outer trimmer edges matched to 95% and 2% OARs, respectively, and (b) trimmer thicknesses sufficiently thick to stop 99% of 20‐MeV electrons. Subsequently, five modifications reduced designed trimmer weights while maintaining acceptable in‐field flatness and out‐of‐field IEC leakage dose. Table 5 shows the cumulative trimmer weight reductions were 45.7% and 51.5% for the 10 × 10 and 20 × 20‐cm^2^ applicators, respectively. Design Modification 5 (determination of trimmer thickness and outer bevel shape) was the most effective, reducing 10 × 10 and 20 × 20‐cm^2^ applicator trimmer weight by 31.3% and 30.8%, respectively. Second most, Design Modification 1 (beveling outer trimmer edges) reduced 10 × 10 and 20 × 20‐cm^2^ applicator trimmer weights by 14.8% and 13.8%, respectively.

### 4.B Weight and mean leakage dose of current and new applicator designs

Table 7 compares trimmer weight and mean leakage dose of the current and new 10 × 10 and 20 × 20‐cm^2^ applicators, showing the new weights reduced by 1.79 kg (32%) and 3.27 kg (39%), respectively, exceeding our design goals. This reduction came at no cost to beam flatness (±3% along major axes; ±4% along diagonal axes) and insignificant cost to out‐of‐field mean leakage dose. The new 10 × 10‐cm^2^ design had less leakage dose at 7 MeV, approximately equal leakage dose at 13 MeV, and slightly greater leakage dose at 20 MeV. The new 20 × 20‐cm^2^ design had slightly greater mean leakage doses (0.12%, 0.06%, and 0.18%) for the 7, 13, and 20 MeV beams, respectively; however, all were well below IEC limits (c.f. Table 6).

**Table 7:** Trimmer weights and mean leakage doses, calculated per IEC criteria,^4^ compared for current^1^, ^2^ and newly designed Elekta 10 × 10 and 20 × 20‐cm^2^ applicators

Applicator	Design	Trimmer weight	Mean leakage dose (% of D_max_)		
			7 MeV	13 MeV	20 MeV
10 × 10 cm^2^	Current	5.52 kg	0.70%	0.65%	0.99%
	New design	3.73 kg	0.58%	0.63%	1.03%
20 × 20 cm^2^	Current	8.36 kg	0.67%	0.69%	0.93%
	New design	5.09 kg	0.79%	0.75%	1.11%

### 4.C Utilizing results for new electron collimation system design

Our 10 × 10 and 20 × 20‐cm^2^ applicator designs (trimmers and X‐ray jaw settings) provide the basis for a new Elekta electron collimation system with lighter applicators, including 6 × 6, 14 × 14, and 25 × 25‐cm^2^ applicators for 6‐20 MeV beams. New applicator sizes retain design parameters from the 10 × 10 and 20 × 20‐cm^2^ applicators (i.e., *z*‐positions, materials, inner edge divergence angles, outer edge bevel forming fluence matching OARs, and trimmer thicknesses) except for OARs at each trimmers inner edge; the 6 × 6 and 25 × 25‐cm^2^ applicators used the same OARs as the 10 × 10 and 20 × 20‐cm^2^ applicators, respectively, for a 6‐MeV beam. The 14 × 14‐cm^2^ applicator OARs were linearly interpolated between the 10 × 10 and 20 × 20‐cm^2^ OARs for a 6‐MeV beam. X‐ray jaw positions for each energy were determined by linearly interpolating the OARs at the upper trimmer inner edge between the 6‐MeV value and 55% at 20 MeV for all applicators (c.f. Table 8).

**Table 8:** OARs (calculated using pencil‐beam theory) at inner edge of trimmers for new applicator designs. Lower and middle trimmer OARs at 6 MeV; upper trimmer OARs interpolated between 6 and 20‐MeV values

	6 × 6 cm^2^	10 × 10 cm^2^	14 × 14 cm^2^	20 × 20 cm^2^	25 × 25 cm^2^
Lower trimmer inner edge matching OAR at 6 MeV	95.0%	95.0%	92.5%	89.0%	89.0%
Middle trimmer inner edge matching OAR at 6 MeV	96.0%	96.0%	94.0%	91.0%	91.0%
Upper trimmer inner edge matching OAR at 6 MeV	96.5%	96.5%	95.0%	93.0%	93.0%
Upper trimmer inner edge matching OAR at 20 MeV	55.0%	55.0%	55.0%	55.0%	55.0%

Calculated weights of these newly designed trimmers for the five Elekta applicators are compared with weights of current applicators and trimmers in Table 9. These results illustrate that our new collimation system offer potentially significantly lighter applicators, e.g., the 25 × 25‐cm^2^ applicator presently weighs 13.4 kg (29.5 lb), whereas a new applicator with equal non‐trimmer weight should weigh 9.5 kg (20.9 lb). The reduced applicator trimmer weights should result in considerably easier applicator handling by the radiotherapy team.

**Table 9:** Comparison of weights (kg) of current with improved‐design Elekta applicators

Applicator	Current elekta		New design
	Applicator	Trimmers only	Trimmers only
6 × 6 cm^2^	7.0	4.75	2.84
10 × 10 cm^2^	7.7	5.52	3.73
14 × 14 cm^2^	9.1	6.71	4.27
20 × 20 cm^2^	10.9	8.36	5.09
25 × 25 cm^2^	13.4	10.00	6.07

### 4.D Sensitivity of new design to X‐ray jaw settings

Currently, Elekta allows jaw adjustments of ±3 cm from factory‐specified settings (at isocenter) for each applicator‐energy combination, usually done during initial accelerator configuration to meet flatness specifications; however, for matched beams, we recommend identical jaw settings because of their impact on other quantities, particularly dose output. Flatness failures or other differences might require modifications to beam tuning and/or the dual scattering foil system.

To study how modifying jaw positions from designed positions impacts in‐field flatness and out‐of‐field leakage dose, a cursory MC study was performed at 7, 13, and 20 MeV.^2^ In‐plane and cross‐plane, X‐ray jaws were modified ±0.5 cm, corresponding to a ±1.0 cm (±1.2) shift at isocenter of the in‐plane (cross‐plane) jaws. Results at all energies showed that moving either jaw 0.5 cm inward caused flatness failure (0‐1.5% low) along the direction of jaw movement and diagonals. Moving either jaw 0.5 cm outward increased the off‐axis dose, but never above 103%, but it increased mean leakage dose by as much as 0.1% at 7 and 13 MeV and 0.3% at 20 MeV. Only 20‐MeV values, 1.40% and 1.44% for moving the in‐plane and cross‐plane jaws, respectively, exceeded IEC‐specified limits (1.34%). Moving either jaw 0.5 cm inward decreased mean leakage dose by as much as 0.1% at 7 and 13 MeV and 0.2% at 20 MeV.^2^

To allow the occasional benefit of small adjustments (e.g., ±1 cm at isocenter) from factory‐specified jaw settings, such “robustness” can be achieved by increasing the width of the constant‐thickness portion of the upper trimmer, which slightly increases the weight. For the 20 × 20‐cm^2^ applicator, the estimated cost is 0.4 kg cm^−1^, which should allow applicator trimmer weights to remain well below both targeted and existing Elekta applicator weights.

### 4.E Further investigation of new collimation system

The prototype collimation system was designed for the MLCi2 treatment head. However, Elekta's Agility treatment head has replaced (a) cross‐plane jaws having curved inner edges with MLC, increasing inner edge area, and (b) in‐plane jaws having diverging inner edges with curved ones. Both alterations could generate increased leakage dose.^1^ Hence, MC calculations should be performed for our prototype 10 × 10 and 20 × 20‐cm^2^ applicators using the Agility treatment head to evaluate in‐field flatness and out‐of‐field leakage dose, allowing for any necessary design adjustments.

## 5 CONCLUSIONS

This study demonstrated a process for designing an Elekta electron collimation system having significantly lighter applicators. 10 × 10 and 20 × 20‐cm^2^ applicators were designed with trimmer weights of 3.73 and 5.09 kg, respectively, well below current weights (5.52 and 8.36 kg, respectively) and our design goals (5.00 and 7.10 kg, respectively). Based on MC calculations at 7, 13, and 20 MeV, both applicator designs produce acceptable in‐field flatness and out‐of‐field leakage. These results have been used to design a new collimations system for 6–20 MeV electron beams with 6 × 6 to 25 × 25‐cm^2^ applicators.

The design of the new electron collimation system (X‐ray jaw settings and applicators) for the Elekta Infinity (MLCi2 treatment head) has been validated by fabricating 10 × 10 and 20 × 20‐cm^2^ prototype applicators and measuring dose. Results of that study confirmed acceptable in‐field flatness and out‐of‐field leakage dose^2^ and will be reported subsequently.

## CONFLICTS OF INTEREST

This research was funded in part through a research agreement with Elekta Limited.
